# Inflammatory Markers in Children and Adolescents with Functional Somatic Disorders: A Systematic Review

**DOI:** 10.3390/children11050549

**Published:** 2024-05-03

**Authors:** Anne Sofie Hansen, Charlotte Ulrikka Rask, Karen Hansen Kallesøe

**Affiliations:** 1 Department of Child and Adolescent Psychiatry, Aalborg University Hospital, 9000 Aalborg, Denmark; 2 Department of Clinical Medicine, Aalborg University, 9000 Aalborg, Denmark; 3 Department of Child and Adolescent Psychiatry, Aarhus University Hospital Psychiatry, 8200 Aarhus N, Denmark; charrask@rm.dk (C.U.R.); karekall@rm.dk (K.H.K.); 4 Department of Clinical Medicine, Aarhus University, 8200 Aarhus N, Denmark

**Keywords:** functional somatic disorders, low-grade inflammation, cytokines, children, adolescents

## Abstract

Functional somatic disorders (FSDs) are common in children and adolescents. Recent findings suggest that low-grade inflammation has a role in the development and maintenance of pediatric FSDs. This systematic review included studies with original data on systemic inflammatory markers in children and adolescents with an FSD compared to individuals without an FSD. The literature search identified 1374 articles. After assessment, a total of 15 studies met the inclusion criteria. In total, 41 serum or plasma cytokines were assayed in a population of 696 children and adolescents. Altered cytokine levels in patients with FSDs were reported in 12 studies, whereas three studies found no significant differences when comparing patients with FSDs and controls. The cytokine levels were significantly elevated in nine studies (i.e., IL-2, IL-6, IL-8, IL-12 (p70), CRP, hsCRP, IP-10, MCP-1, sTIM-3, sCD25 and TNF-α). The findings indicate that inflammatory response may have a role in the pathophysiology of pediatric FSDs. However, the included studies showed limited quality with potential risk of bias, small study populations and a narrow spectrum of included FSDs, which limits the generalizability of the results. To further explore the potential link between inflammatory markers and pediatric FSDs, future research using a longitudinal study design is recommended.

## 1. Introduction

Functional somatic disorders (FSDs) are defined by the presence of one or multiple persistent physical symptoms or characteristic symptom patterns that cannot be attributed to a known well-defined somatic or psychiatric disease. The term encompasses a wide range of diagnoses, such as fibromyalgia, chronic pain, irritable bowel syndrome (IBS) and chronic fatigue syndrome (CFS) [1]. Mild transient physical symptoms are common among children and adolescents; still, the exact number suffering from FSDs remains unknown. A recent meta-analysis found a prevalence rate of 3.3% for FSDs in children and adolescents, and the prevalence is possibly increasing [2,3]. At this severe end of the spectrum, the symptoms typically result in significant disruption of the daily life of the child or adolescent with school absenteeism and social withdrawal and moreover often lead to high levels of health service use [4,5]. There is a substantial risk that symptoms sustain into adulthood, and furthermore, FSDs in childhood and adolescence are associated with adverse physical and mental health outcomes later in life [6,7]. 

FSDs are thought to arise in a complex interaction between biological and psychosocial factors [8]. Although the etiology is still not fully understood, it is suggested to involve different patterns of dysregulation of the stress system, including activation of the immune-inflammatory system. These mechanisms are thought to be triggered by continuing, cumulative or overwhelming stress, potentially resulting in a subtle but chronic state of immune-inflammatory activation, which is termed low-grade inflammation. Low-grade inflammation might activate other stress-system components, such as the hypothalamic–pituitary–adrenal axis, subsequently inducing increased bodily arousal contributing to the generation and maintenance of functional somatic symptoms [9,10,11]. 

The aforementioned inflammatory response, a biological response to an infection or non-infectious process, is brought on by a group of inflammatory markers secreted by immune cells. The main group of systemic inflammatory markers is cytokines, which are protein mediators of the immune system produced by both leukocytes and non-immune cells. Cytokines can be classified depending upon their role in the inflammatory process as pro- or anti-inflammatory, while some cytokines serve as both [12]. Common pro-inflammatory cytokines are IL1-alpha, IL1-beta, IL6, CRP and TNF-alpha, whereas IL-1ra, IL-4, IL-10, IL-11, and IL-13 are common anti-inflammatory cytokines [12]. 

A connection between inflammatory markers and several FSDs has been found in adults; for instance, a study found that levels of high-sensitivity CRP (hsCRP) were significantly elevated for both patients with CSF and patients with fibromyalgia compared to healthy controls [13]. Likewise, another study found that an increased expression of inflammatory markers is associated with a variety of chronic pain conditions, including fibromyalgia, which in turn can lead to an increased release or upregulation of pro-inflammatory cytokines [14]. Considering another important type of FSD, namely IBS, a study found that the pro-inflammatory cytokines TNF-α and IL-17 were significantly higher in adult patients with this disorder compared to healthy controls, suggesting a possible role for low-grade inflammation also in the pathogenesis of IBS [15]. 

It has been proposed that this relationship between FSDs and low-grade inflammation in adults might also be present in children and adolescents with FSDs [16]. A recent review focused on the local inflammatory response in pediatric IBS and suggested a role of low-grade inflammation in the pathophysiology and symptom generation [17]. The high prevalence of children and adolescents presenting with functional somatic symptoms and the multifaceted and not well-understood pathophysiology of FSDs propose a great need for further investigation of this topic. A profile of systemic circulating inflammatory markers in children and adolescents with a broad spectrum of FSDs could potentially generate a better understanding of the complex pathophysiology, ultimately leading to an improvement in early diagnostics and the development of better and more targeted treatment in the future. Thus, the aim of this review is to investigate the possible association between systemic inflammatory markers and FSDs in children and adolescents. 

## 2. Materials and Methods

### 2.1. Review Protocol and Search Strategy

The current systematic review was conducted in accordance with the guidelines set out in the Preferred Reporting Items for Systematic Reviews and Meta-Analyses (PRISMA) [18]. The study information was registered a priori on 11 October 2023 in the Open Science Framework (OSF) registry (https://doi.org/10.17605/OSF.IO/32WNJ, accessed on 11 October 2023). The PICO framework was used to establish the search strategy for the study question [19]. A systematic literature search was performed in the PubMed, PsycINFO and Embase databases. The literature search included both free-text words and identified search terms in MeSH in PubMed, Thesaurus in PsycINFO and Emtree in Embase. The PubMed search string can be found in Supplementary Materials (Table S1). The web-based software program Covidence was used to manage and organize the selection of articles. The final literature search included publications dating up to 23 November 2023.

### 2.2. Eligibility Criteria, Study Selection and Data Collection Process

Studies were considered eligible for data extraction if they met the following inclusion criteria: (1) children or adolescents (aged 0–17 years, both included), (2) patients diagnosed with an FSD, (3) measurement of systemic inflammatory markers, (4) studies including a control group without an FSD, and (5) studies written in English. The exclusion criteria were (1) animal experiments, (2) in vitro experiments, (3) case studies, (4) studies measuring inflammatory response exclusively in feces, biopsies, and urine (i.e., not systemic markers). 

Duplicate references were eliminated, and initial screening was performed based on title and/or abstract. Potentially relevant articles were screened based on the full text. The identification of studies was carried out by two independent evaluators (ASH and KHK), and a third evaluator (CUR) was consulted to resolve discrepancies. All evaluators were experienced clinicians and researchers regarding FSDs in children and adolescents. A kappa estimate of the initial agreement between the two evaluators was calculated for the full text screening. To ensure accurate participant diagnosis, the evaluators assessed that the criteria used to define the FSD diagnoses were within the scope of this study based on the International Classification of Disorders (ICD) and the Diagnostic and Statistical Manual of Mental Disorders (DSM) [20].

Data were extracted from the included studies by the two independent evaluators (ASH and KHK) using a structured data collection form. The data were collated in an Excel spreadsheet and included information on study design, participant characteristics, outcome details regarding the measured inflammatory markers and the results of the comparison between cases and controls. Disagreements were solved through discussion or by consulting the third evaluator (CUR). Finally, a qualitative synthesis of the included data was conducted to summarize and assess the results. A meta-analysis was not applied due to heterogenic data and risk of bias when comparing the cytokine levels across the studies. 

### 2.3. Quality Assessment of Studies

The quality of the included studies was assessed using the Newcastle–Ottawa scale (NOS) of quality to identify biases within studies [21]. Three broad perspectives of case-control and cohort studies are assessed with the NOS using a star system: (1) the selection of the study groups, (2) the comparability of the groups, and (3) the ascertainment of outcome of interest. The total maximum score of these 3 subsets is 9. The quality assessment was conducted independently by two evaluators (ASH and KHK) using the specific NOS criteria (see Supplementary Information S2). A third evaluator (CUR) was consulted to determine final quality scores if discrepancies were not resolved. In the current review a score ≥ 7 was used to categorize high-quality studies based on previously applied cut-offs [22]. 

## 3. Results

### 3.1. Study Selection

**Figure 1:**
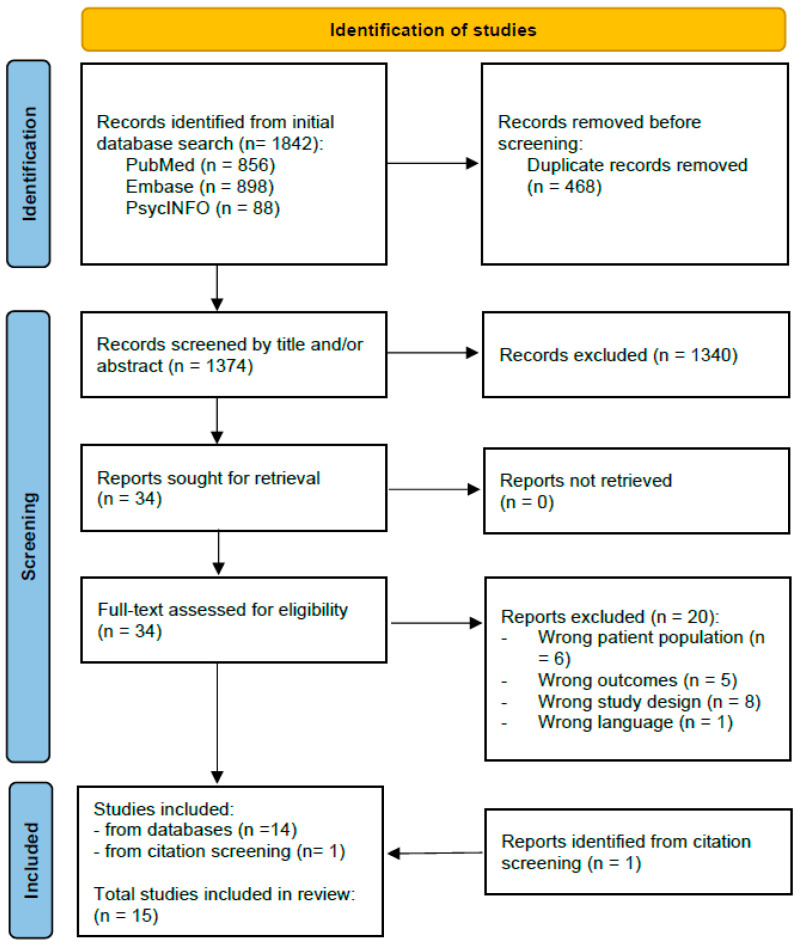
PRISMA flow diagram of the study selection process.

A total of 1842 records were identified in the literature search (PubMed *n* = 856, Embase *n* = 898, and PsycINFO *n* = 88). After duplicate removal, 1374 records were screened based on title and/or abstract. A total of 34 reports were assessed after full-text retrieval, of which 20 reports were excluded, and 14 were determined to fulfill the requirements for inclusion (Kappa of initial agreement = 0.87). Finally, a citation screening of the included studies revealed one further study, leading to the inclusion of a total of 15 studies in the review. The selection process is displayed in Figure 1. 

### 3.2. Study Characteristics

The study characteristics are outlined in Table 1. The included studies were published between 2012 and 2023. The number of cases in the studies ranged from 9 to 100, and a total of 696 cases were included in this review when accounting for overlapping study populations in five of the included reports [16,23,24,25,26]. The cases were defined by CFS in 11 studies and functional gastrointestinal disorders (FGID) were identified in four studies. 

A total of 611 controls were included in this review when considering overlapping study populations [16,23,24,25,26]. The controls were defined as healthy controls in 11 studies [16,23,24,25,26,27,28,29,30,31,32], individuals recovered after Epstein–Barr virus (EBV) or infectious mononucleosis (IM) infection in four studies [24,33,34,35], with one study also including a group of healthy controls [24], and children and adolescents with an asymptomatic parasitic infection in a single study [36]. Three studies used the matching of controls to cases on factors like age, sex, Tanner stage and social class [33,34,35].

**Table 1:** Characteristics of the included studies.

Author (Year, Country)	Study Design	Cases (Definition, Number, Age, Sex)	Controls (Definition, Number, Age, Sex)
**Chronic Fatigue Syndrome (CFS)**			
Al-Rawaf et al. (2019, The Kingdom of Saudi Arabia) [27]	Case-control	CFS *n* = 100 Age: Mean = 16.5 (SD: 2.1) Sex: F = 62 (62%)	Healthy controls *n* = 50 Age: Mean = 16.4 (SD: 2.1) Sex: F = 31 (62%)
Broderick et al. (2012, Canada) [33]	Cohort study (prospective following EBV infection)	CFS *n* = 9 Age: Mean = NR. Range: 12–18 Sex: F = 9 (100%)	Recovered after EBV infection *n* = 12 Age: NR Sex: F = 12 (100%)
Fevang et al. (2021, Norway) [23]	Case-control	CFS *n* = 91 Age: Mean 17.4 (SD: 1.5) Sex: F = 67 (73.6%)	Healthy controls *n* = 70 Age: Mean = 17.0 (SD: 1.8) Sex: F = 44 (62.9%)
Jason et al. (2021, USA) [34]	Cohort study (prospective: before IM infection and after IM infection)	CFS *n* = 47 (severe-CFS = 18; CFS = 29) Age: severe-CFS: Mean = 18.8 (SD: 0.5); CFS: Mean = 18.9 (SD: 0.9) Sex: severe-CFS: F = 61.1%; CFS: F = 74.2%.	Recovered after IM infection *n* = 58 Age: Mean = 18.7 (SD: 2.6) Sex: F = 56%
Jason et al. (2023, USA) [28]	Case-control	CFS *n* = 68 (severe-CFS: 45; CFS: 23) Age: NR (total participants: Mean = 13.8 (SD: NR)) Sex: NR (total participants: F = 57.5%)	Healthy controls *n* = 43 Age: NR (total participants: Mean = 13.8 (SD:NR)) Sex: NR (total participants: F = 57.5%)
Kristiansen et al. (2019, Norway) [24]	Cohort study (prospective following EBV infection)	CFS *n* = 91 Age: Mean 17.4 (SD: 1.5) Sex: F = 67 (73.6%)	Healthy controls *n* = 70 Age: Mean 17.0 (SD: 1.8) Sex: F = 44 (62.9%) EBV non-fatigued *n* = 104 Age: Mean 17.4 (SD 1.7) Sex: F = 60 (57.7%)
Nguyen et al. (2017, Norway) [29]	Case-control	CFS *n* = 29 Age: Mean = 15.1 (SD: 1.4) Sex: F = 18 (62%)	Healthy controls *n* = 18 Age: Mean = 14.7 (SD: 1.4) Sex: F = 11 (61%)
Russell et al. (2016, Canada) [35]	Cohort study (prospective following IM infection)	CFS *n* = 18 Age: Mean = 16.3 (SD: 1.3) Sex: F = 100%	Recovered after IM infection *n* = 24 Age: Mean = 15.8 (SD: 1.67) Sex: F = 100%
Sulheim et al. (2014, Norway) [16]	Case-control	CFS *n* = 120 Age: Mean = 15.4 (SD: NR. Range: 12–18) Sex: F = 86 (71.7%)	Healthy controls *n* = 68 Age: Mean = 15.1 (SD: 1.6) Sex: F = 46 (67.6%)
Wyller et al. (2015, Norway) [25]	Case-control	CFS *n* = 120 Age: Mean = 15.4 (SD: 1.6) Sex: F = 86 (71,7%)	Healthy controls *n* = 68 Age: Mean = 15.1 (SD: 1.6) Sex: F = 46 (68%)
Wyller et al. (2017, Norway) [26]	Case-control	CFS *n* = 120 Age: Mean = 15.4 (SD: 1.6) Sex: F = 88 (73%)	Healthy controls *n* = 68 Age: Mean = 15.1 (SD: 1.6) Sex: F = 46 (68%)
**Functional gastrointestinal disorders (FGID)**			
Hua et al. (2011, Taiwan) [30]	Case-control	IBS *n* = 35 Age: Mean = 13.5 (SD: NR) Sex: F = 15 (42.9%)	Healthy controls *n* = 25 Age: Mean = 12.5 (SD: NR) Sex: F = 11 (44%)
Hua et al. (2013, Taiwan) [31]	Case-control	IBS *n* = 94 Age: Mean = 14.2 (SD: 3.1) Sex: F = 49 (52.1%)	Healthy controls *n* = 102 Age: Mean = 12.3 (SD: 5.1) Sex: F = 61 (59.8%)
Myint et al. (2021, Malaysia) [36]	Case-control	Recurrent abdominal pain (RAP) *n* = 10 Age: Mean = NR. Range: 7–12 Sex: NR.	With asymptomatic parasitic infections *n* = 17 Age: Mean = NR. Range: 7–12. Sex: NR
Sheikh Sajjadieh et al. (2012, Ukraine) [32]	Case-control (population living 60–90 km from Chernobyl exposed to radiation)	IBS *n* = 75 (divided into 3 groups by Age: (1) 4–9 years, (2) 10–13 years, (3) 14–18 years) Age: By group: (1) Mean = 6.4 (SD: 1.8), (2) Mean = 11.4 (SD: 1.0), (3) Mean = 14.0 (SD: 1.0) Sex: NR	Healthy controls *n* = 20 Age: Mean = 10.2 (SD: 3.3) Sex: NR

Abbreviations: CFS: chronic fatigue syndrome; EBV: Epstein–Barr virus; F: female; FSD: functional somatic disorder; *n*: number; IBS: irritable bowel syndrome; IM: infectious mononucleosis; NR: not reported; SD: standard deviation.

### 3.3. Level of Inflammatory Markers

The included studies investigated a broad range of inflammatory markers varying from 1 to 27 assayed cytokines. The results are displayed in Table 2. Across studies, a total of 41 cytokines were included covering the following: bFGF, CRP, eotaxin, G-CSF, GM-CSF, hsCRP, IFN-c, IFN-γ, IL-1α, IL-1β, IL1-ra, IL-2, IL-4, IL-5, IL-6, IL-7, IL-8, IL-9, IL-10, IL-12 (p70), IL-13, IL-15, IL-17, IL-17α, IL-23, IP-10, Ltα, MCP-1, MIP-1a, MIP-1b, PDGF-BB, RANTES, sCD25, sTIM-3, TNF, TNF-α, TNF-β, TGF-β1, TGF-β2, TGF-β3, and VEGF. The cytokines IL-6, IL-10 and TNF were most frequently assayed, and each appeared in more than seven studies [25,27,28,29,30,31,33,34,35,36]. A total of three studies reported no statistically significant difference regarding all assayed cytokines when comparing the cases and controls [25,26,29]. Thus, most studies found altered levels of cytokines in the FSD cases, and nine studies reported statistically significant elevated levels of cytokines in the FSD cases concerning the following specific inflammatory markers: IL-2, IL-6, IL-8, IL-12 (p70), CRP, hsCRP, IP-10, MCP-1, sTIM-3, sCD25 and TNF-α. [16,23,24,27,32,33,34,35,36]. A total of eight studies reported decreased levels of cytokines in the FSD cases regarding the following: IL-5, IL-6, IL-10, IL-13, IL-23, hsCRP and IFN-γ [24,30,31,32,33,34,35,36].

### 3.4. Quality Assessment

The included studies displayed quality scores from 3 to 6 out of a maximum score of 9 as displayed in Table 3. None of the included studies were considered high-quality research studies when assessing the study design using the NOS. Regarding the selection of the cases, the studies lacked consensus regarding the criteria used to determine the CFS diagnosis and used a broad range of definitions, whereas the Rome criteria for IBS were used in three out of the four studies investigating cases with FGID [30,31,32,37]. Potential bias was especially observed regarding the comparability of cases and controls with none of the included studies explicitly stating that the controls had no history of FSD or controlling for psychiatric comorbidity. Eight studies controlled for relevant covariates, i.e., physical activity and/or body mass index (BMI) [16,23,24,25,26,27,29,35]. Finally, when regarding exposure, all studies reported the measured concentrations of the inflammatory markers and used the same method for measuring the inflammatory markers in cases and controls, and two studies reported non-response rates (i.e., participants not completing the blood sample collection) [27,34]. 

## 4. Discussion

This review aimed to summarize existing published data on systemic inflammatory markers in children and adolescents with FSDs compared to controls. In total, 15 studies were identified covering 696 pediatric cases with FSDs only confined to CFS or FGID. A list of 41 serum or plasma cytokines was assayed in the included studies. Altered cytokine levels in children and adolescents with FSDs were reported in 12 studies, whereas only three studies found no significant difference when comparing the FSD cases and controls. The studies, which reported significantly altered cytokine levels in children and adolescents with FSD, displayed both elevated and decreased levels of the assayed cytokines. The reported elevated cytokines are all considered pro-inflammatory when regarding the existing literature [12,38]. However, the reported decreased cytokines capture both pro-inflammatory cytokines (i.e., IL-6, IL-23, hsCRP and IFN-γ) and anti-inflammatory cytokines (i.e., IL-5, IL-10, IL-13) [12,38]. This makes it difficult to firmly conclude whether the inflammatory response is expected to be increased or decrease in children and adolescents with FSDs. Still, the main part of the reported altered cytokine levels mediates a pro-inflammatory response, indicating a process of low-grade inflammation in children and adolescents with FSDs. 

When regarding the existing evidence on adults with FSDs, a recent systematic review by Blundell et al. found no significant differences in cytokine levels between adult CFS cases and healthy controls, except transforming growth factor-beta (TGF-β), across the majority of the 38 included studies [39]. Still, a number of studies on adults with other FSDs (e.g., fibromyalgia and chronic pain) point in the direction of an association between inflammation response and FSD [13,14,40]. Furthermore, a large register-based cohort study on adult cases with a broad range of FSDs found that former infections leading to hospital contacts were associated with a higher risk of having an FSD, and the number of prior infections increased the risk of an FSD, also indicating an association between FSDs and severe infections [41]. 

In pediatric FSDs, the TRAILS study by Joncker et al. investigated a population of adolescents with non-neurological functional somatic symptoms [42]. The study did not include a control group but compared the measured levels of hsCRP with the reported burden of functional somatic symptoms. A significant association between hsCRP levels and the level of functional somatic symptoms was shown, indicating a role for inflammatory processes associated with the severity of symptoms in pediatric FSD. Furthermore, two studies from Australia by Kozlowska et al. and McInnis et al. investigated children with functional neurological disorders as well as children with chronic functional pain and measured CRP levels [43,44]. The studies did not include control groups but compared reference ranges on CRP levels. Both studies reported an upward shift in CRP levels in the FSD cases, suggesting an activation of the inflammatory response system, also indicating a role for inflammatory processes in pediatric FSD. 

A skewing of the immune system with altered cytokine production has been suggested to be linked to long-term stress exposure causing an activation of the hypothalamic–pituitary–adrenal (HPA) axis and the inflammatory system. In a state of chronic stress, prolonged activation of the HPA axis with elevated production of the stress hormone cortisol can cause impairment of the immune system’s sensitivity to cortisol, resulting in elevated levels of inflammatory markers [45]. The influence of chronic stress on the HPA axis may change over time, transitioning from heightened to diminished activity, ultimately leading to inadequate regulation of inflammation [46]. The relationship between cortisol levels and FSDs has been investigated in prior studies, indicating an altered cortisol response in FSDs [47,48]. However, long-term stress can also inhibit the inflammatory response system mediated by elevated levels of stress hormones, causing a shift in the balance of certain T-lymphocyte types (e.g., Th1, Th2 and Th17) resulting in lowered levels of cytokines [49]. Thus, the interplay between the stress system and inflammatory response is a complex process, and the development of FSDs may be mediated by an imbalance of both elevated and lowered levels of different cytokines, as also displayed with the results of this current review. Furthermore, the heterogenic alterations in cytokine levels observed in this review may reflect a varying pathophysiology due to the different bio-psycho-social underlying factors involved in the development and maintenance of FSDs, as also displayed in this review including both post-infectious and post-radiation conditions. 

To summarize, this review outlined the existing knowledge and displayed a possible association between the level of inflammatory markers and FSDs in children and adolescents. The noteworthy strengths of this paper are the comprehensive systematic literature search and the inclusion of studies reporting on FSD cases compared to controls. However, the evidence level is limited by the quality of the included studies as well as the lack of generalizability of the data due to a narrow spectrum of investigated FSDs as well as different types and numbers of assayed inflammatory markers in these studies. Regarding the measured level of the inflammatory markers, we decided not to conduct a meta-analysis, since the heterogeneity in applied methods for measuring the cytokine levels made it difficult to compare the results across the studies. None of the included studies reported data on the usage of anti-inflammatory medication, although patients with FSDs may occasionally use non-steroidal anti-inflammatory drugs (NSAID)s to relieve pain. Furthermore, the small sample size of the studies and the lack of matching regarding control groups may lead to bias, and it is important to interpret the results with caution. 

Future research could aim to apply a consistent methodology in case-control designs investigating a wide range of cytokines and testing the association with subgroups of pediatric FSDs. The studies identified in this systematic review only investigated children and adolescents with either CFS or FGID. Future studies should include a broad spectrum of FSDs to examine levels of systemic inflammatory markers across FSDs. Furthermore, a longitudinal study of cytokine levels before and after the onset of FSDs in children and adolescents, as well as after remission, could provide knowledge regarding the relationship between circulating cytokines and the development and maintenance of FSDs. Possible confounders such as comorbid psychiatric and somatic conditions, physical activity, weight as well as medication should be accounted for to avoid potential bias.

## 5. Conclusions

This systematic review aimed to outline the existing body of evidence regarding the level of inflammatory markers in children and adolescents with FSDs. Altered levels of inflammatory markers in pediatric FSDs were reported in most of the included studies, indicating a role for the inflammatory response system in the pathophysiology of pediatric FSDs. However, the review demonstrates that the quality of the existing studies is limited, and further research is recommended to explore the possible role of inflammatory processes in the development and maintenance of pediatric FSDs. 

## Figures and Tables

**Table 2:** Main results of the included studies.

Author (Year)	Serum/Plasma Inflammatory Markers		
	Elevated in FSD Cases:	Lower in FSD Cases:	No Significant Difference Compared to Controls/Other Results Reported:
**Chronic Fatigue Syndrome (CFS)**			
Al-Rawaf et al. (2019) [27]	IL-6, TNF-α		
Broderick et al. (2012) [33]	IL-8, IL-2	IL-23, IL-5	IL-1a, IL-1b, IL-4, IL6, IL10, IL12 (p70), IL13, IL15, IL17, IFN-γ, TNF-α, TNF-β
Fevang et al. (2021) [23]	CRP, IP-10, MCP-1, sTIM-3, sCD25		RANTES, TGF-β1
Jason et al. (2021) [34]	At 6-month follow-up: IL-12 (p70)	At baseline: IL-6, IL-13, IL-5 At 6-month follow-up: IL-13	At baseline and follow-up: IL-1α, IL-1β, IL-2, IL-4, IL-8, IL-10, IL-13, IL-17α, IL-23, IFN-γ, TNF-α, TNF-β
Jason et al. (2023) [28]			The following cytokine networks were associated with the FSD cases: (1) IL-12p70, IL-17A and IFN-γ; (2) IL-17A and IL-23; (3) IL-6 and IL-8
Kristiansen et al. (2019) [24]	hsCRP (compared to non-fatigued EBV controls)	hsCRP (compared to healthy controls)	
Nguyen et al. (2017) [29]			CRP, IL-1β, IL-6, TNF
Russell et al. (2016) [35]	IL-8	IL-23	IL1α, IL1β, IL2, IL4, IL5, IL6, IL10, IL12, IL13, IL15, IL17, IFN-γ, TNF-α, Ltα
Sulheim et al. (2014) [16]	CRP		CRP (when controlling for depressive symptoms)
Wyller et al. (2015) [25]			IL-1b, IL1-ra, IL-2, IL-4, IL-5, IL-6, IL-7, IL-8, IL-9, IL-10, IL-12 (p70), IL-13, IL-15, IL-17, eotaxin, bFGF, G-CSF, GM-CSF, IFN-c, IP-10, MCP-1, MIP-1a, MIP-1b, PDGF-BB), RANTES, TNF, VEGF
Wyller et al. (2017) [26]			TGF-β (TGF-β1, TGFβ2, TGF-β3)
**Functional gastrointestinal disorders (FGID)**			
Hua et al. (2011) [30]		IL-10	TNF-a, IL-6
Hua et al. (2013) [31]		IL-10	
Myint et al. (2021) [36]	TNF-α	IL-10, IL-6	
Sheikh Sajjadieh et al. (2012) [32]	IL-4	IFN-γ	

Abbreviations: bFGF: basic fibroblast growth factor; CRP: C-reactive protein; FSD: functional somatic disorder; G-CSF: granulocyte colony-stimulating factor; GM-CSF: granulocyte–macrophage colony-stimulating factor; hsCRP: high-sensitive C-reactive protein; IFN: interferon; IL: interleukin; Ltα: lymphotoxin alpha; MCP-1: monocyte chemoattractant protein-1; MIP: macrophage inflammatory protein; PDGF-BB: platelet-derived growth factor-BB; RANTES: Regulated upon Activation, Normal T Cell Expressed and Presumably Secreted; sCD25: alpha-chain of IL-2 receptor; sTIM-3: soluble T cell immunoglobulin domain 3; TNF: tumor necrosis factor; TGF: transforming growth factor; VEGF: vascular endothelial growth factor.

**Table 3:** Quality assessment of included studies according to Newcastle–Ottawa scale (NOS) ^a^.

Author (Year)	Selection:				Comparability:		Exposure:			Total:
	1	2	3	4	5	6	7	8	9	
**Chronic Fatigue Syndrome (CFS)**										
Al-Rawaf et al. (2019) [27]	-	*	*	-	-	*	*	*	*	6
Broderick et al. (2012) [33]	*	*	*	-	-	-	*	*	-	5
Fevang et al. (2021) [23]	*	*	*	-	-	*	*	*	-	6
Jason et al. (2021) [34]	*	*	*	-	-	-	*	*	*	6
Jason et al. (2023) [28]	*	*	*	-	-	-	*	*	-	5
Kristiansen et al. (2019) [24]	*	*	*	-	-	*	*	*	-	6
Nguyen et al. (2017) [29]	*	*	*	-	-	*	*	*	-	6
Russell et al. (2016) [35]	*	-	*	-	-	*	*	*	-	5
Sulheim et al. (2014) [16]	*	*	*	-	-	*	*	*	-	6
Wyller et al. (2015) [25]	*	*	*	-	-	*	*	*	-	6
Wyller et al. (2017) [26]	*	*	*	-	-	*	*	*	-	6
**Functional Gastrointestinal Disorders (FGID)**										
Hua et al. (2011) [30]	*	*	-	-	-	-	*	*	-	4
Hua et al. (2013) [31]	*	*	-	-	-	-	*	*	-	4
Myint et al. (2021) [36]	*	*	-	-	-	-	*	*	-	4
Sheikh Sajjadieh et al. (2012) [32]	*	-	-	-	-	-	*	*	-	3

^a^ The NOS used for the quality assessment is outlined in Supplementary Information S2. * A star was awarded if the requirements of the item was rated to be fulfilled.

## Data Availability

No new data were created or analyzed in this study. Data sharing is not applicable to this article.

## Supplementary Materials

The following supporting information can be downloaded at https://www.mdpi.com/article/10.3390/children11050549/s1; Table S1 displays the search string used to conduct the database literature search; The NOS used for the quality assessment is outlined in Supplementary Information S2.

## References

[1] Burton C., Fink P., Henningsen P., Löwe B., Rief W. (2020). Functional somatic disorders: Discussion paper for a new common classification for research and clinical use. BMC Med..

[2] Vesterling C., Schütz-Wilke J., Bäker N., Bolz T., Eilts J., Koglin U., Rademacher A., Goagoses N. (2023). Epidemiology of Somatoform Symptoms and Disorders in Childhood and Adolescence: A Systematic Review and Meta-Analysis. Health Soc. Care Community.

[3] Van Geelen S.M., Hagquist C. (2016). Are the time trends in adolescent psychosomatic problems related to functional impairment in daily life? A 23-year study among 20,000 15–16 year olds in Sweden. J. Psychosom. Res..

[4] O’Sullivan P.B., Beales D.J., Smith A.J., Straker L.M. (2012). Low back pain in 17 year olds has substantial impact and represents an important public health disorder: A cross-sectional study. BMC Public. Health.

[5] Rask C.U. (2012). Functional somatic symptoms in 5–7 year old children: Assessment, prevalence and co-occurrence. Dan. Med. J..

[6] Bohman H., Jonsson U., Päären A., von Knorring L., Olsson G., von Knorring A.-L. (2012). Prognostic significance of functional somatic symptoms in adolescence: A 15-year community-based follow-up study of adolescents with depression compared with healthy peers. BMC Psychiatry.

[7] Holley A.L., Law E.F., Zhou C., Murphy L., Clarke G., Palermo T.M. (2013). Reciprocal longitudinal associations between pain and depressive symptoms in adolescents. Eur. J. Pain..

[8] Kangas M., Kallesoe K.H., Rask C.U. (2020). Functional Somatic Syndromes (FSS) in Children and Adolescents: Conceptual, Measurement and Treatment Issues. Z. Psychol..

[9] Irwin M.R. (2011). Inflammation at the intersection of behavior and somatic symptoms. Psychiatr. Clin. N. Am..

[10] Turnbull A.V., Rivier C.L. (1999). Regulation of the hypothalamic-pituitary-adrenal axis by cytokines: Actions and mechanisms of action. Physiol. Rev..

[11] Kozlowska K., Scher S., Helgeland H. (2020). The Immune-Inflammatory System and Functional Somatic Symptoms. Functional Somatic Symptoms in Children and Adolescents: A Stress-System Approach to Assessment and Treatment.

[12] Wautier J.-L., Wautier M.-P. (2023). Pro- and Anti-Inflammatory Prostaglandins and Cytokines in Humans: IA Mini Review/I. Int. J. Mol. Sci..

[13] Groven N., Fors E.A., Reitan S.K. (2019). Patients with Fibromyalgia and Chronic Fatigue Syndrome show increased hsCRP compared to healthy controls. Brain Behav. Immun..

[14] Montague K., Malcangio M. (2017). The therapeutic potential of targeting chemokine signalling in the treatment of chronic pain. J. Neurochem..

[15] Choghakhori R., Abbasnezhad A., Hasanvand A., Amani R. (2017). Inflammatory cytokines and oxidative stress biomarkers in irritable bowel syndrome: Association with digestive symptoms and quality of life. Cytokine.

[16] Sulheim D., Fagermoen E., Winger A., Andersen A.M., Godang K., Müller F., Rowe P.C., Saul J.P., Skovlund E., Øie M.G. (2014). Disease mechanisms and clonidine treatment in adolescent chronic fatigue syndrome: A combined cross-sectional and randomized clinical trial. JAMA Pediatr..

[17] Di Nardo G., Cremon C., Staiano A., Stanghellini V., Borrelli O., Strisciuglio C., Romano C., Mallardo S., Scarpato E., Marasco G. (2023). Role of inflammation in pediatric irritable bowel syndrome. Neurogastroenterol. Motil..

[18] Page M.J., McKenzie J.E., Bossuyt P.M., Boutron I., Hoffmann T.C., Mulrow C.D., Shamseer L., Tetzlaff J.M., Akl E.A., Brennan S.E. (2021). The PRISMA 2020 statement: An updated guideline for reporting systematic reviews. BMJ.

[19] Da Costa Santos C.M., de Mattos Pimenta C.A., Nobre M.R.C. (2007). The PICO strategy for the research question construction and evidence search. Rev. Lat. Am. Enfermagem.

[20] Creed F. (2023). Progress in understanding functional somatic symptoms and syndromes in light of the ICD-11 and DSM-5. World Psychiatry.

[21] Luchini C., Stubbs B., Solmi M., Veronese N. (2017). Assessing the quality of studies in meta-analyses: Advantages and limitations of the Newcastle Ottawa Scale. World J. Metaanal.

[22] Gianfredi V., Mazziotta F., Clerici G., Astorri E., Oliani F., Cappellina M., Catalini A., Dell’osso B.M., Pregliasco F.E., Castaldi S. (2024). Climate Change Perception and Mental Health. Results from a Systematic Review of the Literature. Eur. J. Investig. Health Psychol. Educ..

[23] Fevang B., Wyller V.B.B., Mollnes T.E., Pedersen M., Asprusten T.T., Michelsen A., Ueland T., Otterdal K. (2021). Lasting Immunological Imprint of Primary Epstein-Barr Virus Infection With Associations to Chronic Low-Grade Inflammation and Fatigue. Front. Immunol..

[24] Kristiansen M.S., Stabursvik J., O’Leary E.C., Pedersen M., Asprusten T.T., Leegaard T., Osnes L.T., Tjade T., Skovlund E., Godang K. (2019). Clinical symptoms and markers of disease mechanisms in adolescent chronic fatigue following Epstein-Barr virus infection: An exploratory cross-sectional study. Brain Behav. Immun..

[25] Wyller V.B., Sørensen Ø., Sulheim D., Fagermoen E., Ueland T., Mollnes T.E. (2015). Plasma cytokine expression in adolescent chronic fatigue syndrome. Brain Behav. Immun..

[26] Wyller V.B., Nguyen C.B., Ludviksen J.A., Mollnes T.E. (2017). Transforming growth factor beta (TGF-β) in adolescent chronic fatigue syndrome. J. Transl. Med..

[27] Al-Rawaf H.A., Alghadir A.H., Gabr S.A. (2019). MicroRNAs as Biomarkers of Pain Intensity in Patients With Chronic Fatigue Syndrome. Pain. Pract..

[28] Jason L.A., Gaglio C.L., Furst J., Islam M., Sorenson M., Conroy K.E., Katz B.Z. (2023). Cytokine network analysis in a community-based pediatric sample of patients with myalgic encephalomyelitis/chronic fatigue syndrome. Chronic Illn..

[29] Nguyen C.B., Alsøe L., Lindvall J.M., Sulheim D., Fagermoen E., Winger A., Kaarbø M., Nilsen H., Wyller V.B. (2017). Whole blood gene expression in adolescent chronic fatigue syndrome: An exploratory cross-sectional study suggesting altered B cell differentiation and survival. J. Transl. Med..

[30] Hua M.-C., Lai M.-W., Kuo M.-L., Yao T.-C., Huang J.-L., Chen S.-M. (2011). Decreased interleukin-10 secretion by peripheral blood mononuclear cells in children with irritable bowel syndrome. J. Pediatr. Gastroenterol. Nutr..

[31] Hua M.-C., Chao H.-C., Yao T.-C., Lai M.-W., Huang J.-L. (2013). Investigation of interleukin-10 promoter polymorphisms and interleukin-10 levels in children with irritable bowel syndrome. Gut Liver.

[32] Sajjadieh M.R.S., Kuznetsova L., Bojenko V. (2012). Cytokine status in Ukrainian children with irritable bowel syndrome residing in a radioactive contaminated area. Iran. J. Immunol..

[33] Broderick G., Katz B.Z., Fernandes H., Fletcher M.A., Klimas N., Smith A.F., O’gorman M.R., Vernon S.D., Taylor R. (2012). Cytokine expression profiles of immune imbalance in post-mononucleosis chronic fatigue. J. Transl. Med..

[34] Jason L.A., Cotler J., Islam M.F., Sunnquist M., Katz B.Z. (2021). Risks for Developing Myalgic Encephalomyelitis/Chronic Fatigue Syndrome in College Students Following Infectious Mononucleosis: A Prospective Cohort Study. Clin. Infect. Dis..

[35] Russell L., Broderick G., Taylor R., Fernandes H., Harvey J., Barnes Z., Smylie A., Collado F., Balbin E.G., Katz B.Z. (2016). Illness progression in chronic fatigue syndrome: A shifting immune baseline. BMC Immunol..

[36] Myint K., Jacobs K., Myint A.-M., Lam S.K., Lim Y.A.-L., Boey C.C.-M., Hoe S.Z., Guillemin G.J. (2021). Psychological Stresses in Children Trigger Cytokine- and Kynurenine Metabolite-Mediated Abdominal Pain and Proinflammatory Changes. Front. Immunol..

[37] Drossman D.A. (2016). Functional Gastrointestinal Disorders: History, Pathophysiology, Clinical Features, and Rome IV. Gastroenterology.

[38] Harvanová G., Duranková S., Bernasovská J. (2023). The role of cytokines and chemokines in the inflammatory response. Alergol. Pol. Pol. J. Allergol..

[39] Blundell S., Ray K.K., Buckland M., White P.D. (2015). Chronic fatigue syndrome and circulating cytokines: A systematic review. Brain Behav. Immun..

[40] García J.J., Cidoncha A., Bote M.E., Hinchado M.D., Ortega E. (2014). Altered profile of chemokines in fibromyalgia patients. Ann. Clin. Biochem..

[41] Schovsbo S.U., Møllehave L.T., Petersen M.W., Bjerregaard A.A., Eliasen M., Pedersen S.B., Eplov L.F., Kårhus L.L., Fink P., Linneberg A. (2022). Association between infections and functional somatic disorders: A cross-sectional population-based cohort study. BMJ Open.

[42] Jonker I., Schoevers R., Klein H., Rosmalen J. (2017). The association between herpes virus infections and functional somatic symptoms in a general population of adolescents. The TRAILS study. PLoS ONE.

[43] McInnis P.M., Braund T.A., Chua Z.K., Kozlowska K. (2020). Stress-system activation in children with chronic pain: A focus for clinical intervention. Clin. Child. Psychol. Psychiatry.

[44] Kozlowska K., Chung J., Cruickshank B., McLean L., Scher S., Dale R.C., Mohammad S.S., Singh-Grewal D., Prabhuswamy M.Y., Patrick E. (2019). Blood CRP levels are elevated in children and adolescents with functional neurological symptom disorder. Eur. Child. Adolesc. Psychiatry.

[45] Ji D., Francesconi M., Flouri E., Papachristou E. (2022). The role of inflammatory markers and cortisol in the association between early social cognition abilities and later internalising or externalising problems: Evidence from a UK birth cohort. Brain Behav. Immun..

[46] Agorastos A., Pervanidou P., Chrousos G.P., Baker D.G. (2019). Developmental trajectories of early life stress and trauma: A narrative review on neurobiological aspects beyond stress system dysregulation. Front. Psychiatry.

[47] Del Paso G.A.R., Garcia-Hernandez A., Contreras-Merino A.M., Galvez-Sánchez C.M., de la Coba P., Montoro C.I., Davydov D.M. (2024). A two-component model of hair cortisol concentration in fibromyalgia: Independent effects of pain chronicity and severity. Eur. J. Pain..

[48] Nyengaard R., Kallesøe K.H., Rimvall M.K., Ørnbøl E., Wellnitz K.B., Olsen E.M., Wyller V.B.B., Rask C.U. (2024). Hair cortisol and self-perceived stress in adolescents with multi-system functional somatic disorders. BMC Psychiatry.

[49] Assaf A.M., Al-Abbassi R., Al-Binni M. (2017). Academic stress-induced changes in Th1- and Th2-cytokine response. Saudi Pharm. J..

