# Supplementary material for: Inflammatory Markers in Children and Adolescents with Functional Somatic Disorders: A Systematic Review
Source: Children (Basel). 2024 May 3;11(5):549. doi: 10.3390/children11050549 (PMC11119612; doi:10.3390/children11050549)
Supplement: Supplementary file 1 [file children-11-00549-s001.zip › children-2963554-supplementary.pdf]

Supplementary Materials

Table S1. Search string, PubMed

**FUNCTIONAL SOMATIC DISORDERS**	"Somatoform Disorders"[Mesh] OR somatoform disorder*[Title/Abstract] OR somatoform symptom*[Title/Abstract] OR somatoform pain*[Title/Abstract] OR somatoform compliant*[Title/Abstract] OR conversion disorder*[Title/Abstract] OR functional neurological disorder*[Title/Abstract] OR bodily distress disorder*[Title/Abstract] OR bodily distress symptom*[Title/Abstract] OR Dissociative Disorders[Mesh] OR dissociative disorder*[Title/Abstract] OR Chronic Fatigue Syndrome[Mesh] OR fatigue syndrome*[Title/Abstract] OR chronic fatigue*[Title/Abstract] OR persistent fatigue*[Title/Abstract] OR prolonged fatigue*[Title/Abstract] OR Fibromyalgia[Mesh] OR fibromyalgi*[Title/Abstract] OR "Psychophysiologic Disorders"[Mesh] OR psychophysiologic disorder*[Title/Abstract] OR psychosomatic disorder*[Title/Abstract] OR psychosomatic symptom*[Title/Abstract] OR psychosomatic complaint*[Title/Abstract] OR psychosomatic illness*[Title/Abstract] OR Tension Type Headache[Mesh] OR functional headach*[Title/Abstract] OR non-specific headach*[Title/Abstract] OR nonspecific headach*[Title/Abstract] OR tension headach*[Title/Abstract] OR tension-type headach*[Title/Abstract] OR tensiontype headach*[Title/Abstract] OR recurrent headach*[Title/Abstract] OR idiopathic headach*[Title/Abstract] OR chronic headach*[Title/Abstract] OR Chronic Pain[Mesh] OR chronic pain*[Title/Abstract] OR chronic limb pain*[Title/Abstract] OR chronic back pain*[Title/Abstract] OR chronic neck pain*[Title/Abstract] OR chronic musculoskeletal pain*[Title/Abstract] OR chronic benign pain*[Title/Abstract] OR functional pain*[Title/Abstract] OR psychogenic pain*[Title/Abstract] OR longstanding pain*[Title/Abstract] OR persistent pain*[Title/Abstract] OR idiopathic pain*[Title/Abstract] OR chronic widespread pain*[Title/Abstract] OR "Colonic Diseases, Functional"[Mesh] OR recurrent abdominal pain*[Title/Abstract] OR functional abdominal pain*[Title/Abstract] OR functional gastrointestinal symptom*[Title/Abstract] OR functional gastrointestinal disorder*[Title/Abstract] OR functional gastrointestinal pain*[Title/Abstract] OR chronic abdominal pain*[Title/Abstract] OR chronic abdominal complaint*[Title/Abstract] OR chronic abdominal symptom*[Title/Abstract] OR irritable bowel syndrome*[Title/Abstract] OR Medically Unexplained Symptoms[Mesh] OR unexplained symptom*[Title/Abstract] OR unexplained complaint*[Title/Abstract] OR unexplained illness*[Title/Abstract] OR unexplained physical symptom*[Title/Abstract] OR unexplained somatic complaint*[Title/Abstract] OR medically unexplained physical symptom*[Title/Abstract] OR nonspecific musculoskeletal symptom*[Title/Abstract] OR non-specific musculoskeletal symptom*[Title/Abstract] OR nonspecific musculoskeletal complaint*[Title/Abstract] OR non-specific musculoskeletal complaint*[Title/Abstract] OR nonspecific musculoskeletal disorder*[Title/Abstract] OR non-specific musculoskeletal disorder*[Title/Abstract] OR nonspecific musculoskeletal pain*[Title/Abstract] OR non-specific musculoskeletal pain*[Title/Abstract] OR idiopathic musculoskeletal pain*[Title/Abstract] OR musculoskeletal complaint*[Title/Abstract] OR functional somatic[Title/Abstract] OR

	somatic symptom disorder*[Title/Abstract] OR persistent physical symptom*[Title/Abstract] OR functional somatic syndrome*[Title/Abstract] OR non-cardiac chest pain*[Title/Abstract] OR noncardiac chest pain*[Title/Abstract] OR hyperventilation syndrome*[Title/Abstract] OR somatization disorder*[Title/Abstract] OR "somatic symptom and related disorder*"[Title/Abstract] OR "somatic symptoms and related disorder*"[Title/Abstract] OR SSRD[Title/Abstract]
**INFLAMMATION**	(Inflammation*[Title/Abstract] OR (Inflammation[MeSH Terms]) OR (Inflammatory response*[Title/Abstract]) OR (Inflammatory marker*[Title/Abstract]) OR (Inflammation marker*[Title/Abstract]) OR (Inflammatory mediator*[Title/Abstract]) OR (Inflammation mediator*[Title/Abstract]) OR (Cytokine*[Title/Abstract]) OR (Low-grade inflammation*[Title/Abstract]) OR (Proinflammatory marker*[Title/Abstract]) OR (Proinflammatory mediator*[Title/Abstract]) OR (Pro-inflammatory marker*[Title/Abstract]) OR (Pro-inflammatory mediator*[Title/Abstract]) OR (Inflammation mediators[MeSH Terms]) OR (Cytokines[MeSH Terms]))

Supplementary Information S2

Quality assessment

Review: **Inflammatory Response in Children and Adolescents with Functional Somatic Disorders**

NEWCASTLE - OTTAWA QUALITY ASSESSMENT SCALE: CASE CONTROL / COHORT STUDIES

(Note: A study can be awarded a maximum of one star for each numbered item within the Selection and Exposure categories. A maximum of two stars can be given for Comparability).

- **Selection:**
  1) Is the case definition adequate?
     a) yes, with independent validation ***** (the case definition scores positively if accepted diagnostic criteria for FSD were used and an adequate explanation of how this was assessed)
     b) no description

  2) Representativeness of the cases
     a) consecutive or obviously representative series of cases * (if the study included both males and females, the proportion of males to females was representative of the population studies of FSD (i.e. F>M) or were not subject to selection bias. Studies were considered to be subject to selection bias when patients recruited from only one setting, e.g. secondary care clinic only)
     b) potential for selection biases or not stated

  3) Selection of Controls:
     a) community controls * (if the controls were from the same community/geographical location as the cases)
     b) hospital controls
     c) no description

  4) Definition of Controls
     a) no history of disease (endpoint) * (the study had to explicitly state that controls had no history of FSD)
     b) no description of source

- **Comparability:**
  5+6) Comparability of cases and controls on the basis of the design or analysis:
     a) study controls for psychiatric comorbidity *
     b) study controls for physical activity and/or obesity *

- **Exposure:**
  7) Ascertainment of outcome (inflammatory markers):
     a) Secure record (The measure of inflammatory marker concentration: the paper had to explicitly state the concentration of the measured inflammatory markers to score a star for eligibility point) *

b) structured interview where blind to case/control status * (laboratory blinded to samples)
c) interview not blinded to case/control status
d) written self report or medical record only
e) no description

8) Same method of ascertainment for cases and controls:
a) yes *
b) no

9) Non-Response rate
a) same rate for both groups *
b) non respondents described
c) rate different and no designation
